# 3D printed collagen/silk fibroin scaffolds carrying the secretome of human umbilical mesenchymal stem cells ameliorated neurological dysfunction after spinal cord injury in rats

**DOI:** 10.1093/rb/rbac014

**Published:** 2022-02-24

**Authors:** Chong Chen, Hai-Huan Xu, Xiao-Yin Liu, Yu-Sheng Zhang, Lin Zhong, You-Wei Wang, Lin Xu, Pan Wei, Ya-Xing Chen, Peng Liu, Chen-Ru Hao, Xiao-Li Jia, Nan Hu, Xiao-Yang Wu, Xiao-Song Gu, Li-Qun Chen, Xiao-Hong Li

**Affiliations:** 1 Academy of Medical Engineering and Translational Medicine, Tianjin University, 92 Weijin Road, Nankai District, Tianjin 300072, China; 2 Tianjin Key Laboratory of Neurotrauma Repair, Pingjin Hospital Brain Center, Characteristic Medical Center of People’s Armed Police Forces, 220 Chenglin Road, Dongli District, Tianjin 300162, China; 3 Department of Neurosurgery, West China Medical School, West China Hospital, Sichuan University, Guoxue Lane 37, Wuhou District, Chengdu, Sichuan 610041, China; 4 National Engineering Research Center for Biomaterials, College of Biomedical Engineering, Sichuan University, 29 Wangjiang Road, Wuhou District, Chengdu, Sichuan 610064, China; 5 Department of Hematology, The First Affiliated Hospital of Chengdu Medical College, 278 Baoguang Avenue middle section, Xindu District, Chengdu, Sichuan 610500, China; 6 Medical Psychology Section, Hubei General Hospital of Armed Police Force, 475 Minzhu Road, Wuchang District, Wuhan, Hubei 430071, China; 7 Department of Neurosurgery, The First People’s Hospital of Long Quan yi District, Yuyang Road Yihe 3 Group, Longquanyi District, 201 Cheng Du, Si Chuan 610000, China

**Keywords:** spinal cord injury, secretome, mesenchymal stem cells, 3D printing, collagen, silk fibroin

## Abstract

Although implantation of biomaterials carrying mesenchymal stem cells (MSCs) is considered as a promising strategy for ameliorating neural function after spinal cord injury (SCI), there are still some challenges including poor cell survival rate, tumorigenicity and ethics concerns. The performance of the secretome derived from MSCs was more stable, and its clinical transformation was more operable. Cytokine antibody array demonstrated that the secretome of MSCs contained 79 proteins among the 174 proteins analyzed. Three-dimensional (3D) printed collagen/silk fibroin scaffolds carrying MSCs secretome improved hindlimb locomotor function according to the Basso–Beattie–Bresnahan scores, the inclined-grid climbing test and electrophysiological analysis. Parallel with locomotor function recovery, 3D printed collagen/silk fibroin scaffolds carrying MSCs secretome could further facilitate nerve fiber regeneration, enhance remyelination and accelerate the establishment of synaptic connections at the injury site compared to 3D printed collagen/silk fibroin scaffolds alone group according to magnetic resonance imaging, diffusion tensor imaging, hematoxylin and eosin staining, Bielschowsky’s silver staining, immunofluorescence staining and transmission electron microscopy. These results indicated the implantation of 3D printed collagen/silk fibroin scaffolds carrying MSCs secretome might be a potential treatment for SCI.

**Figure:**
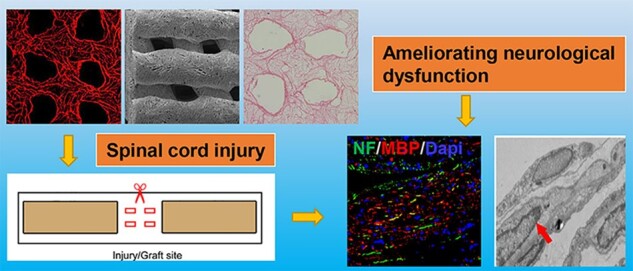

## Introduction

Spinal cord injury (SCI) usually results in loss of motor, sensory and autonomic functions [1–6]. Non-permissive microenvironment inhibits central nervous system (CNS) axon regeneration after injury [7–10]. Glial and neuronal cell death, axon loss, demyelination, inflammation, glial scar formation, necrotic cavity, neurotrophic factor deficiency and other pathological changes occur after SCI, resulting in hostile microenvironment leading to permanent functional dysfunction, neurological deficits and limited regenerative capacity [11–14]. Despite extensive research, there is still no effective and definitive clinical treatment for SCI, especially, complete SCI [13]. Recent studies show that neurotrophic factor-based tissue engineering technology holds promise as a strategy for treating SCI [3, 10, 15–19]. With the insight from the combination of three-dimensional (3D) printing technology and tissue engineering technology, tissue engineering technology has created a new era of tissue repair [11, 20–22].

Cell transplantation can be used for the treatment of CNS diseases including SCI. Many studies have shown that mesenchymal stem cells (MSCs) can promote functional recovery in multifarious CNS models [23]. Increasing evidence indicates that MSCs can also ameliorate neurogenesis [24–26]. MSCs transplantation in clinical is limited by immunologic rejection, tumorigenicity and ethical concerns [17]. Many studies have indicated that paracrine factors are conducive to the therapeutic effects of MSCs [27–31]. Increasing evidence has demonstrated that secretome (ST) played a critical role in the MSCs-induced neural regeneration [28, 32–35].

The ST of MSCs contains many proteins composed of growth factors and cytokines, as well as vesicle parts of exosomes and microvesicles [36, 37]. The nature of the essential components of the therapeutic MSCs ST remains subtle. Sart *et al*. [38] indicated that the ST of MSCs could facilitate the endogenous secretion of the extracellular matrix of neural progenitor aggregates, as well as secreting transforming growth factor-β1 (TGF-β1), brain-derived neurotrophic factor and fibroblast growth factor-2 to accelerate cell adhesion and proliferation of the cells. Three-dimensional printed biomaterial not only controlled the release rate of ST *in vivo*, but also supported the adhesion, survival, migration and differentiation of neural stem cells (NSCs) [39, 40].

A wide variety of biomaterials are widely used to facilitate SCI repair. By bridging the two ends of the SCI area, biomaterials can reconnect the nerve tissue in the injured area, hinder scar formation, reduce inflammatory processes, rebuild nerve conduction circuits and prevent spinal neuron apoptosis, and ultimately ameliorate locomotion function after SCI [41, 42]. The basic parameters of biomaterials, such as biocompatibility, biodegradability, permeability and porosity, are critical in the study of neural tissue engineering [43]. Many natural and synthetic polymers are used as scaffold materials. Collagen, as a natural extracellular matrix component, is widely used in various tissue engineering studies because of its excellent biocompatibility, biodegradability, low immunogenicity and cell adhesion [11, 22, 44]. Some limitation, such as poor mechanical properties, weak manipulability and rapid degradation of collagen hinder its clinical application [45]. Silk fibroin possesses mechanical strength, significant elasticity and environmental stability. Silk fibroin is currently used as a biomaterial for soft tissue engineering and reconstruction [46, 47]. The addition of silk fibroin in collagen can make up for the shortcomings of using a collagen scaffold alone.

Good mechanical properties of biomaterials should guide axon regeneration before being completely biodegraded, and be strong enough to withstand stress from the spine and surrounding muscles [48]. The rapid biodegradation rate and low mechanical strength of certain natural materials have hindered their use as tissue engineering materials, and 3D printing technology is required to ameliorate their mechanical properties and bridge the two ends of the SCI area and obtain the favorable effect for SCI treatment. Furthermore, the application of tissue engineering is limited by the preparation technology. In SCI animal experiments, the diameter of the scaffold implanted at the injury site is only about 3 mm [49]. Traditional manufacturing technologies, such as particle leaching technology, extrusion technology and thermal compression, are actually difficult to achieve in the large-scale manufacturing of scaffolds with accurate internal microstructures. As a solid free-form manufacturing technology in tissue engineering, the latest advent of 3D printing provides a novel and feasible method to ameliorate the properties of scaffolds [49, 50]. During the 3D printing process, the print head prepares a 3D composite scaffold containing a series of 2D layers. Printing parameters can be continuously adjusted and optimized on the computer workstation [51, 52]. Three-dimensional printing technology integrates mechanical engineering, computer-aided design (CAD), reverse engineering technology, layered manufacturing technology and numerical control technology, so that it has high accuracy and can achieve a multi-level structure with the coexistence of large holes and micro holes. Therefore, this technology can print scaffolds with complex internal structures, control the size of pores and ensure the biological activity of biomaterials.

In the present study, we try to fabricate 3D printed collagen/silk fibroin scaffolds adsorbed with the ST of human umbilical mesenchymal stem cells (HUCMSCs) and aimed to investigate the repair effect of the implanted scaffold as well as their potential to reconstruct neural networks after SCI.

## Materials and methods

### Isolation, culture and analysis of HUCMSCs

According to existing methods [53, 54], HUCMSCs were isolated from the Wharton’s jelly of the umbilical cord provided by the Obstetrics and Gynecology Department of Characteristic Medical Center of People’s Armed Police Forces, and the pregnant woman and her family members signed the informed consent. After cutting the umbilical cord Wharton’s glue, it was treated with collagenase and 0.25% trypsin. The cell suspension was then obtained by filtering the digested mixture through a 100-μm filter. The extracted cells were cultured in an incubator with a volume fraction of 5% CO_2_ at 37°C, and the culture medium was changed once after 3 days. After 70–80% cell fusion, the cells were digested with 0.25% trypsin and subcultured. An inverted phase-contrast microscope was used to observe the morphology of HUCMSCs. The immunophenotype of HUCMSCs (p3-generation) was analyzed using immunofluorescence staining [55]. Rabbit polyclonal antibody against CD90 (1:200, Bioss, Beijing, China) and mouse polyclonal antibody against CD105 (1:200, Bioss, Beijing, China) were used as primary antibodies. The secondary primary antibodies were used: Alexa Fluor 568-conjugated (1:1000, Invitrogen, Carlsbad, CA, USA) and Oregon Green 488-conjugated secondary antibodies (1:1000, Invitrogen, Carlsbad, CA, USA). The images were observed and captured by using a fluorescence microscope (Leica TCS SP5, Germany). According to the existing method [53, 54], the flow cytometer was performed to analyze the surface antigen of HUCMSC (passage 3). The antibodies used were as follows: CD90, CD105, CD73, CD116, CD19, CD45 and HLA-DR antibodies (Abcam, Cambridge, UK). Cytomics FC500 flow cytometer (BD Biosciences, San Jose, CA) was used to analyze the positive rate of these antigens.

### Isolation and collection of HUCMSCs–ST

The ST derived from HUCMSCs was obtained according to existing methods [56]. To generate the ST, primary HUCMSCs were seeded onto DMEM culture flasks (2 × 10^6^ cells/flask (75 cm^2^) containing 10% fetal bovine serum and cultured for 24 h. The medium was then replaced with serum-free, low-glucose DMEM. The conditioned media was collected after 24 h of culture in serum-free low-glucose DMEM, and then centrifuged at 500 × g for 10 min once, followed by centrifugation at 800 × g for 15 min twice at 4°C. The supernatants were concentrated by ultrafiltration using a Minimate Tangential Flow Filtration (TFF) capsule system (PALL Corporation, Ann Arbor, MI) with a 100 kDa membrane. Subsequently, ST produced by 1 × 10^7^ cells was concentrated to 20 μL. A bicinchoninic acid (BCA) protein assay kit (Beyotime, China) was performed to measure the proteins concentration of ST.

### Cytokine antibody array

An antibody-based cytokine array system (RayBio Human Cytokine Antibody Array C Series 2000, RayBiotech Inc. Norcross, GA) was implemented to analyze a panel of specified proteins in supernatant of HUCMSCs as previously described [56]. In brief, after dilution with blocking buffer (at a ratio of 1:50 (v/v)), the samples were put in a pre-blocked array overnight at 4°C. After subsequent incubation in streptavidin-conjugated peroxidase for 2 h, membranes were exposed to a peroxidase substrate for 5 min, followed by developing on X-ray film. A Kodak ImageStation 4000 M (Eastman Kodak, Rochester, NY) was performed to analyze optical density (OD) with the background subtracted from the edge of the spots. A positive control spot on each array was obtained by normalizing the spot data.

### Fabrication of scaffolds

Silk fibroin was fabricated with silkworm silk (Jiaxing, Zhejiang, China) following the previously reported method [57–59]. Silkworm silk was placed in 0.5% anhydrous sodium carbonate solution (Solarbio Science & Technology Co., Ltd. China) and treated at 98–100°C for 30 min (the operation was repeated three times, the first two times were using tap water, and the third time using ionized water to degumming), and then oven dried at 60°C. The ternary solution of CaCl_2_.CH_3_CH_2_OH.H_2_O (substance concentration ratio of 1:2:8, the amount of each substance used is 188.7 g, 197.54 ml, 244.8 ml) (Solarbio Science & Technology Co., Ltd. China) was prepared at 60°C to dissolve 50 g silk, and then a mixed solution was obtained in 2 h. The mixed solution was cooled, centrifuged in an 8000 r/min centrifuge for 10 min, and the supernatant was taken and placed in a 3500D dialysis bag. Dialysis was performed with flowing tap water for 48 h, deionized water for 1 day, and water was changed once every 2 h. After dialysis, the solution was placed in 40% PEG (molecular weight 20 000 polyethylene glycol, deionized water), concentrated for 7–8 h and concentrated to about 15% in order to obtain an appropriate concentration of silk fibroin solution. Collagen was fabricated by using the fresh bovine tendon according to the previous protocols [58–60]. The fresh bovine tendon was peeled off the outer membrane, crushed and soaked in 0.05 Mtris buffer (Solarbio Science & Technology Co., Ltd. China) for 24 h to obtain the precipitate. An acetic acid solution (North China Pharmaceutical Factory, China) containing pepsin (Solarbio Science & Technology Co., Ltd. China) was then added and the supernatant was collected. The supernatant was added to a 3.5 mol/l NaCL solution, and the salted-out precipitate was collected and dialyzed at 4°C for 5 days to get the collagen gel.

To fabricate scaffolds, the silk fibroin protein and collagen were evenly mixed at a mass ratio of 1:2 for later preparation of 3D printing. For low temperature 3D printing technology, 3D bioprinter (Regenovo Biotechnology Co., Ltd., Hangzhou, China) was implemented to create constructs at –20°C. CAD was used to construct a multi-hole support template. Modeling software (Solidworks Software, Dassault Systèmes, Vélizy, France) was performed to design the 3D printing model for this study, followed by converting the pre-designed STL 3D model into G code. The printing parameters of this study were as follows: nozzle diameter = 160 μm, extrusion speed = 0.17 mm/min, printing speed = 12 mm/s, thickness = 0.3 mm per layer, platform temperature = –20°C. After the collagen/silk fibroin mixed solution was put into the printer cartridge, the material was squeezed out from the printing nozzle under the control of the computer and quickly cooled into a solid form. After printing, the 3D solid molding was placed at –80°C overnight and vacuum freeze-dried for 48–72 h to form 3D collagen/silk fibroin scaffold (3D-C/S) with a stable morphology. Cylinder-shaped 3D-C/S scaffolds (2 mm ×3 mm ×3 mm) were manufactured with a hole punch and sterilized by ^60^Co (Jinpengyuan Irradiation Technology Co., Ltd., Tianjin, China). ST solution was sterilized by using a 0.22 μm filter (Millipore) at 4°C. Then to equilibrium binding, 0.1 g 3D printed collagen/silk fibroin scaffolds were soaked with 20 μL ST solution (200 μg) for 24 h at 4°C under sterile conditions to prepare 3D printed collagen/silk fibroin scaffolds adsorbed with ST derived from HUCMSCs (3D-C/S+ST). D-Hank’s solution was used to wash all scaffolds 30 min before use.

### Determination of scaffold characteristics

As previously stated, ultrastructural observations of scaffolds were performed by using a scanning electron microscope (SEM) (Hitachi, Tokyo, Japan) [22]. The water absorption ratio and the porosity ratio were examined as previously described [22]. The dynamic mechanical properties of the scaffolds are used to represent the mechanical behavior of the support under cyclic load in wet state. The mechanical properties of the scaffolds were measured by Instron 5865 (Instron, Norwood, MA, USA) material testing machine. The scaffold was immersed in PBS (PH = 7.4) at 37°C for 24 h until equilibrium, and the elastic modulus of the compression strain of the three groups of samples was measured. A 0.5 Hz sawtooth waveform was used, the preload was 0.1 N, the increment was 50%, the speed was 100%/min and the cycle was repeated three times. Ten scaffolds were measured in each group of samples, and the mean value and standard deviation (SD) of the obtained data were calculated to obtain the elastic modulus parameters of the scaffolds. An infrared spectrometer (Nicolet 870; Thermo Fisher Scientific, Waltham, MA, USA) was used to analyze Fourier transform infrared spectroscopy of 3D-C/S, 3D-C/S+ST and ST [60]. Differential scanning calorimetry (DSC) (DSC822e differential scanning calorimeter; Mettler Toledo, Greifensee, Switzerland) was carried out to analyze the phase transition temperatures of 3D-C/S, 3D-C/S+ST and ST [60]. The X-ray diffractometer (D8 Advance, Germany) was used to perform crystallization analysis of 3D-C/S, 3D-C/S+ST and ST. The relevant parameters for X-ray diffractometry were as follows: tube voltage = 40 kV, tube current = 30 mA, target material was CuKα (= 0.15406 nm), scanning speed = 5°/min, step width = 0.02° and the diffraction angle range = 5°–90°.

### Seeding of NSCs

NSCs isolated from embryonic Day 14 brains were cultured in the 3D-C/S and 3D-C/S+ST to evaluate the cytocompatibility of scaffolds according to the method previously reported [60, 61]. NSCs (passage 4) were seeded on 3D-C/S and 3D-C/S+ST at a density of 1 × 10^6^/ml. An inverted phase contrast phase-contrast microscope (Nikon, Tokyo, Japan) was performed to observe the length of cell protrusions and angle of cell protrusions of NSCs on 3D-C/S and 3D-C/S+ST at 7 days after co-culture. The morphology and growth of the cells on 3D-C/S and 3D-C/S+ST were observed on Day 7 by using the SEM (Hitachi, Tokyo, Japan). Hematoxylin and eosin (HE) staining was implemented to assess the growth status of NSCs on 3D-C/S and 3D-C/S+ST on Day 7; 3D-C/S and 3D-C/S+ST were put into 96-well plates separately to quantify the cell adhesion rate. Twenty microliters of NSCs were seeded at a density of 5 × 10^6^/ml per scaffold. Cell adhesion rate of NSCs was measured after incubation for 1, 12, 24, 36, 48, 60 and 72 h. The formula for calculating the cell adhesion rate was as follows: cell adhesion rate = (number of adherent cells/number of seeding cells) × 100%.

### *In vitro* NSCs viability

To evaluate the effect of the scaffold on the proliferation of neural stem cells, the viability of neural stem cells is analyzed by Cell Counting Kit-8 (CCK-8) assay (Solarbio Science & Technology Co., Ltd); 100 μL of NSC was seeded on the 3D-C/S and 3D-C/S+ST in each well of the 96-well plate at a density of 1 × 10^9^/L (five duplicate wells per group). At 1, 3, 5 and 7 days after co-culture, 10 μL CCK-8 solution was added to each well, followed by culture for 3 h. A multi-mode microplate reader (BioTek, Synergy2, USA) was implemented to detect the OD value at 450 nm.

### Surgery for spinal cord transection and scaffold implantation

All rats’ experiments conformed to the ‘Policies for the Use of Animals and Humans in Neuroscience Research’ and were approved by the Institutional Animal Care and Use Committee of Logistics University of People’s Armed Police (PAP) (approval No. 2019-1183.6). The experimental procedures for all rats were approved by the Animal Ethics Committee for PAP Research with reference number 36793/68 for ethical approval.

According to the method previously reported [60], a complete transection of the 2 mm spinal cord segment at the T10 spinal cord level was performed on adult female Sprague-Dawley rats (250–280 g) to establish the rat SCI model. Immediately after exact hemostasis, 3D-C/S and 3D-C/S+ST were implanted to fill up the transected gap in the scaffold groups. According to different treatments method, all rats were randomized into four groups: (i) Sham group, (only laminectomy without SCI, *n* = 20); (ii) SCI group, (SCI without any implantation, *n* = 20); (iii) 3D-C/S group, (3D printed collagen/silk fibroin scaffolds were implanted into the transected gap in the rats with spinal cord transection, *n* = 20); (iv) 3D-C/S+ST group, (3D printed collagen/silk fibroin scaffolds adsorbed ST derived from HUCMSCs were implanted into the transected gap in the rats with spinal cord transection, *n* = 20). For postoperative care, the rats received penicillin for infection prevention within 7 days after surgery and manual bladder compression twice daily until reflex bladder control was restored.

### Evaluation of locomotor function

The behavioral assessment on all rats in an open area was carried out by two observers blind to the experimental conditions to evaluate the degree of functional restoration after SCI according to the Basso–Beattie–Bresnahan (BBB) scale at 1 day before the operation, and at 1 day, 1, 2, 3, 4, 6 and 8 weeks after SCI (*n* = 10 for each group). All rats were subjected to an inclined-grid climbing test at 1 day before the operation, and at 1 day, 1, 2, 4, 6 and 8 weeks after SCI (*n* = 10 for each group). Rats could maintain the maximum angle of the board and the ground when the body was held for 5 s in two different positions on the board, and the angle to be measured was obtained. The bilateral hind limb movements of all rats were observed at 8 weeks after SCI.

### Electrophysiological analysis

Neurophysiology monitoring setup was used to evaluate motor evoked potentials (MEP) with evoked potential equipment (Nicolet VikingQuest; Natus Medical Inc., Pleasanton, CA, USA) at 2, 4, 6 and 8 weeks after surgery (*n* = 10 for each group). Changes in the MEP latency and amplitude were observed. MEP was recorded to assess the functional status of motor axonal conduction. The implementation of electrophysiological analysis was based on previously established methods [11, 22]. Briefly, the cortical motor area at the intersection of the coronal and sagittal sutures was selected as the location of the stimulating electrode. The posterior tibial nerve was positioned to record the electrode. The parameters to be set were as follows: the stimulus intensity (46 V), the stimulation frequency (1 Hz) and the pulse width (0.2 ms). The MEP waveform can be obtained from the monitor and the amplitude and latency can be analyzed after electrical stimulation.

### Magnetic resonance imaging and diffusion tensor imaging

To observe the morphology of the SCI area, magnetic resonance imaging (MRI) and diffusion tensor imaging (DTI) were carried out as previously described at 8 weeks after surgery (*n* = 10 for each group) [60]. To evaluate the continuity of the nerve fiber bundles in the spinal cord, diffusion tensor tractography (DTT), the number of regenerated nerve fibers at the injury site and fractional anisotropy (FA) values were obtained, which cannot be examined by MRI [3, 62]. Thirteen FA values were measured at 1.5-mm intervals between the rostral and caudal of each rat by using Trackvis software, followed by drawing FA value distribution map.

### Histological analysis

At 8 weeks after SCI, HE staining and Bielschowsky’s silver staining were performed for pathological examination according to an established method (*n* = 10 for each group) [11, 63–65]. Ten slices were selected from every fifth section of the posterior ventral surface of the spinal cord. After taking photos, Image-Pro Plus software was performed for quantitative statistics. For HE staining, the SCI area (width 2 mm, length 3 mm) was selected to calculate the cavity area. For Bielschowsky’s silver staining, the SCI area (width 2 mm, length 3 mm) was selected to calculate Bielschowsky’s silver staining area.

### Immunofluorescence staining

At 8 weeks after SCI, the rats were transcardially perfused with isotonic saline and then 4% paraformaldehyde. The spinal cord sections were embedded, followed by cryosection longitudinally into 20 μm slices. The primary antibodies were incubated with the samples of the injury/graft site overnight at 4°C (*n* = 10 for each group). The primary antibodies used were as follows: Neurofilament (NF), 1:200; Myelin Basic Protein (MBP), 1:500; PSD95, 1:500; SYP, 1:200 (Abcam, Cambridge, UK). The secondary antibodies (Alexa Fluor 568-conjugated (1:1000, Invitrogen, Carlsbad, CA, USA) and Oregon Green 488-conjugated (1:1000, Invitrogen, Carlsbad, CA, USA)) were applied to the samples and incubated at 37°C for 1 h. A fluorescent microscope (Leica TCS SP5, Germany) was carried out to observe the samples and capture the images. Two areas (0.40 mm × 0.40 mm) at the injury/graft site were selected separately. Image-Pro Plus software (Media Cybernetics) was performed for the quantitative analysis according to an established method [60].

### Transmission electron microscopy

A Philips CM10 electron microscope (Eindhoven, Holland) was used to examine remyelination in the SCI area at Week 8 according to existing methods (*n* = 5 for each group) [66]. The sections at the injury/graft site were cut into ultrathin sections (100 nm thickness) by an ultramicrotome (Reichert E, Co, Vienna, Austria). The number of myelinated axons at the injury site was quantified by selecting at least 10 random fields per sample at ×1500 magnification. The myelinated axons diameter and thickness of myelin sheath at the injury site were measured by using Image-Pro Plus software (Media Cybernetics).

### Statistical analysis

IBM SPSS Statistics 22 was used for statistical analyses. The quantitative data were represented as mean ± SD values. Two sets of data were compared by using an independent-samples *t-test* or two-way analysis of variance. When the *P* values < 0.05, a statistically significant difference was considered.

## Results

### Morphology and characteristic protein identification of HUCMSCs

**Figure 1.**
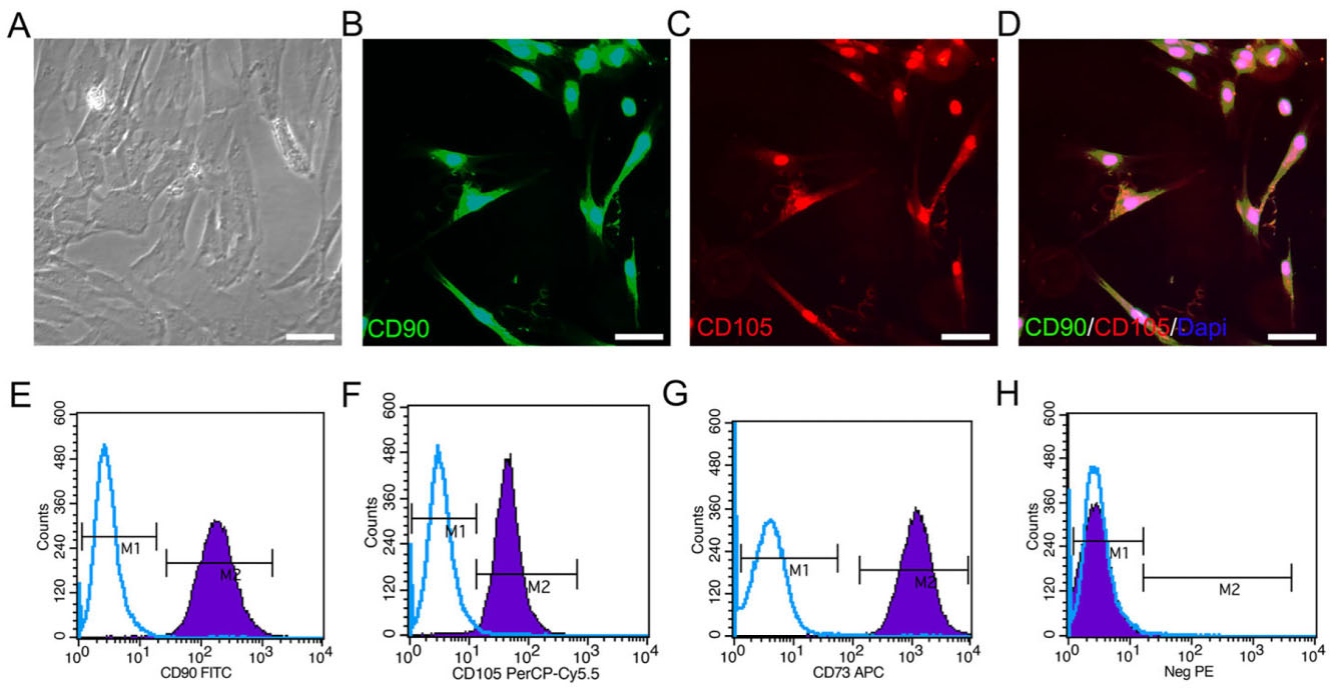
Cell marker analysis of HUCMSCs. (**A**) Typical HUCMSCs morphology at p3-generation under a phase-contrast microscope. (**B**–**D**) Typical images of HUCMSCs stained with CD90 (green) and CD105 (red). (**E**–**H**) HUCMSCs characteristic proteins (CD90, CD105, CD73 and negative control (neg PE) (CD45, CD116, CD19 and HLA-DR)) analyzed by flow cytometry. Scale bars = 50 µm in panels (A–D).

The fusiform HUCMSCs at p3-generation were observed by using a phase-contrast microscope (Fig. 1A). The expression of surface markers CD90 and CD105 in HUCMSCs was detected by a fluorescent microscope (Fig. 1B–D). The results of flow cytometry showed that the expression rates of CD90, CD105 and CD73 were all > 95%, which also proved that HUCMSCs had been successfully extracted and the purity of HUCMSCs could meet the requirements of this experiment (Fig. 1E–H).

### The proteins of HUCMSCs–ST

The concentration of ST, measured by a BCA protein assay kit, was measured to be ∼ 10 mg/ml. To ascertain therapeutic molecular mediators in the HUCMSCs ST, high-density protein arrays were used to analyze the levels of 174 key signaling proteins in ST. The results of the cytokine antibody array indicated that ST contained 79 proteins among the 174 proteins analyzed. The 79 proteins of ST were presented in detail in previously published studies [56].

### Characterization of 3D-C/S+ST

**Figure 2.**
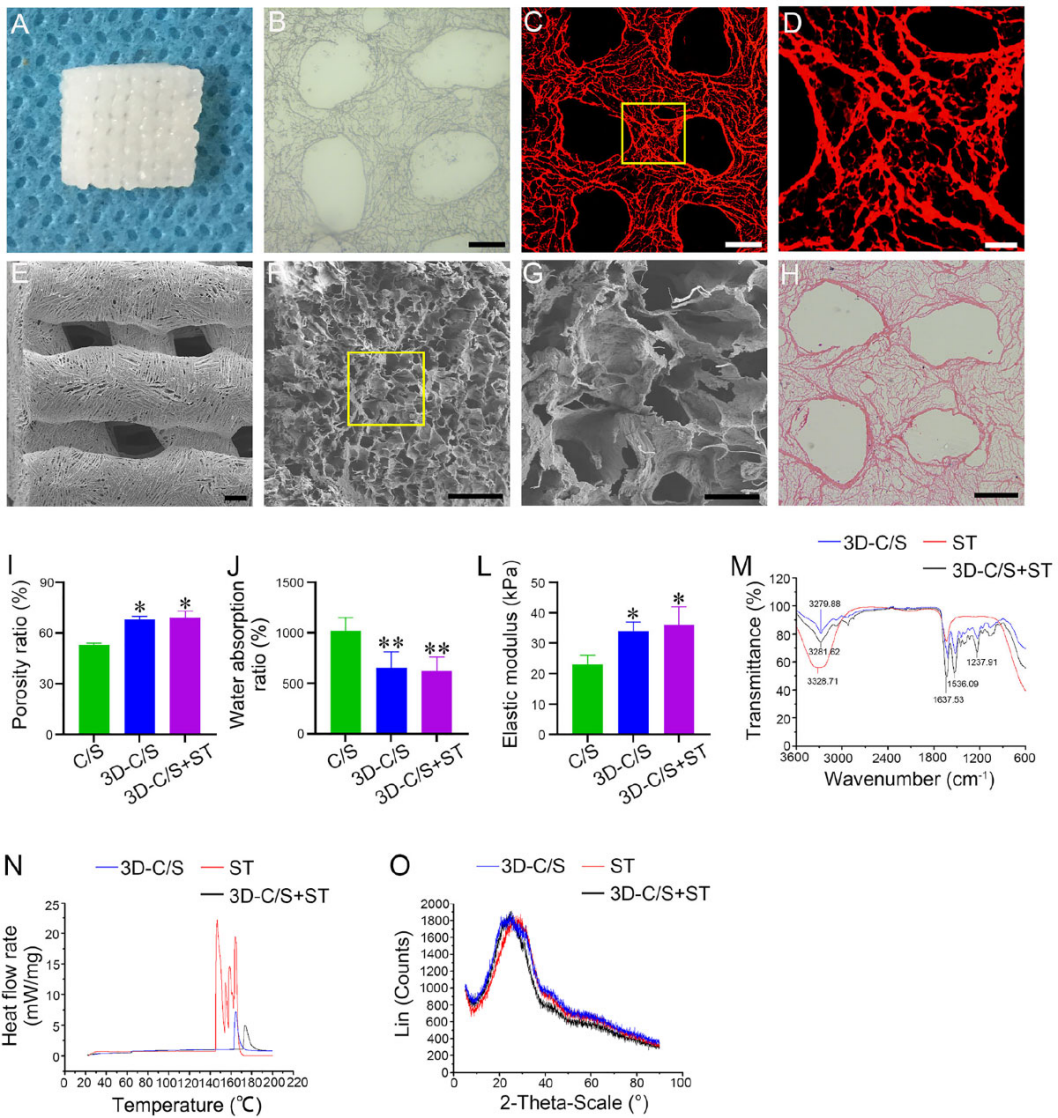
Characterization of scaffold. (**A**) The scaffold was prepared by a 3D bioprinter. (**B**–**H**) The porous microstructure of 3D-C/S+ST under light microscope (B), fluorescent microscope (C and D), SEM (E–G) and HE staining (H). The yellow box regions in (C and F) were enlarged in (D and G), respectively. (**I**–**K**) Porosity ratio, water absorption ratio and elastic modulus of C/S, 3D-C/S and 3D-C/S+ST. (**L**) Infrared spectra examination of 3D-C/S, 3D-C/S+ST and ST. (**M**) DSC examination of 3D-C/S, 3D-C/S+ST and ST. (**N**) X-ray diffraction examination of 3D-C/S, 3D-C/S+ST and ST. Scale bars = 200 µm in panels (B, C, E and H), 50 µm in panels (D and F), 10 µm in panel G. *P <* *0.05, **P <* *0.01 vs C/S.

3D-C/S+ST were prepared using a 3D bioprinter (Fig. 2A); 3D scaffolds were shown to be porous by light microscope (Fig. 2B), fluorescent microscope (Fig. 2C and D), SEM (Fig. 2E–G) and HE staining (Fig. 2H), facilitating cell adhesion and growth. Compared to C/S, the porosity ratio of 3D-C/S (*P *<* *0.05) and 3D-C/S+ST (*P *<* *0.05) was significantly increased, which promoted the diffusion of nutrient solution and tissue formation (Fig. 2I). The water absorption ratio of 3D-C/S (*P *<* *0.01) and 3D-C/S+ST (*P *<* *0.01) was lower compared to C/S, which facilitated the maintenance of ST (Fig. 2J). 3D-C/S (*P *<* *0.05) and 3D-C/S+ST (*P *<* *0.05) exhibited a significant increase in the elastic modulus compared to C/S, which provided support for tissue regeneration (Fig. 2K). 3D-C/S and 3D-C/S+ST showed similar porosity ratio, water absorption ratio and elastic modulus. Infrared spectrum showed that ST was successfully added to 3D printed collagen/silk fibroin scaffolds (Fig. 2L). Through the combination of ST, the absorption peak at 3281.62 cm^−1^ in 3D-C/S+ST distinctly increased compared to that in 3D-C/S alone (Fig. 2L). Moreover, compared with 3D-C/S, the absorption peak at 1536.09 cm^−1^ and 1637.53 cm^−1^ in 3D-C/S+ST were sharper and turned to a lower wave number (Fig. 2L). The results of the infrared spectroscopy indicated that hydrogen bonds between collagen/silk fibroin and ST may be formed. For DSC, Tm was the apex of the endothermic change caused by temperature, which might be caused by the denaturation of ST. The Tm value of 3D-C/S+ST (Fig. 2M) was higher than that of pure ST, which suggested that the interaction between ST and 3D printed collagen/silk fibroin might augmented the stability of ST. X-ray diffraction analysis suggested a sharp diffraction peak at 24.54° was observed and the crystallinity of the sample changed with the amount of silk fibroin added to collagen (Fig. 2N). In this experiment, 3D-C/S and 3D-C/S+ST showed favorable crystallinity, which was beneficial to the formation of the stability of the scaffold and the control of the degradation rate.

### 3D-C/S+ST showed favorable cytocompatibility

**Figure 3.**
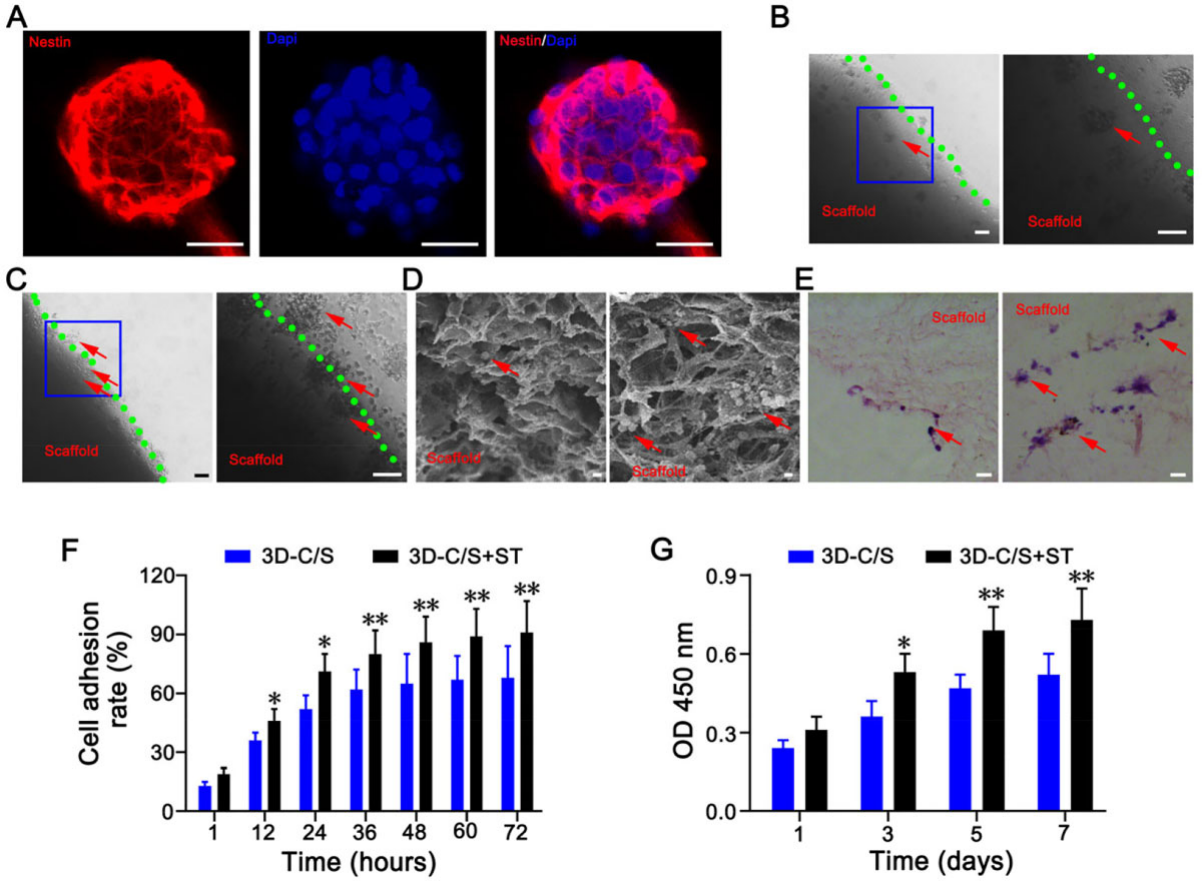
NSCs study of scaffolds. (**A**) Immunofluorescence staining of NSCs-specific marker nestin. (**B** and **C**) Phase-contrast microscope images of NSCs on 3D-C/S (B) and 3D-C/S+ST (C). The blue box regions in the left were enlarged in the right. (**D** and **E**) Representative SEM images (D) and HE staining images (E) reflecting the growth of NSCs on 3D-C/S (left) and 3D-C/S+ST (right). (**F**) Cell adhesion rates of NSCs on 3D-C/S and 3D-C/S+ST after co-culture. (**G**) CCK-8 assay of NSCs on the 3D-C/S and 3D-C/S+ST. All red arrows indicated NSCs. Scale bars = 50 µm in panels (A, B, C and E), 5 µm in panel (D). *P <* *0.05, **P <* *0.01 vs 3D-C/S.

Immunofluorescence staining showed that Nestin was expressed in the neurospheres (Fig. 3A). After 7 days of co-culture, NSCs grew on the surface and in the pores of 3D-C/S+ST and 3D-C/S using phase-contrast microscope, SEM, HE staining and immunofluorescence staining (Fig. 3B–E). Simultaneously, the number of NSCs adhering to 3D-C/S+ST was significantly higher than that of 3D-C/S (Fig. 3B–E). The implantation of 3D-C/S+ST increased cell adhesion rate of NSCs compared to 3D-C/S at the same time point (Fig. 3F). Compared with implanting 3D-C/S, implanting 3D-C/S+ST showed a significant increase in the absorbance of NSCs based on CCK-8 assay (*P *<* *0.05) (Fig. 3G). These results indicated that both 3D-C/S+ST and 3D-C/S scaffolds had cytocompatibility and 3D-C/S+ST group was more conducive to cell growth compared to 3D-C/S group. 3D-C/S+ST and 3D-C/S exhibited good cytocompatibility and therefore have the potential to be implanted *in vivo* for biomedical study.

### HUCMSCs–ST released from the implant of 3D-C/S+ST facilitated locomotor function and electrophysiological activity after SCI

**Figure 4.**
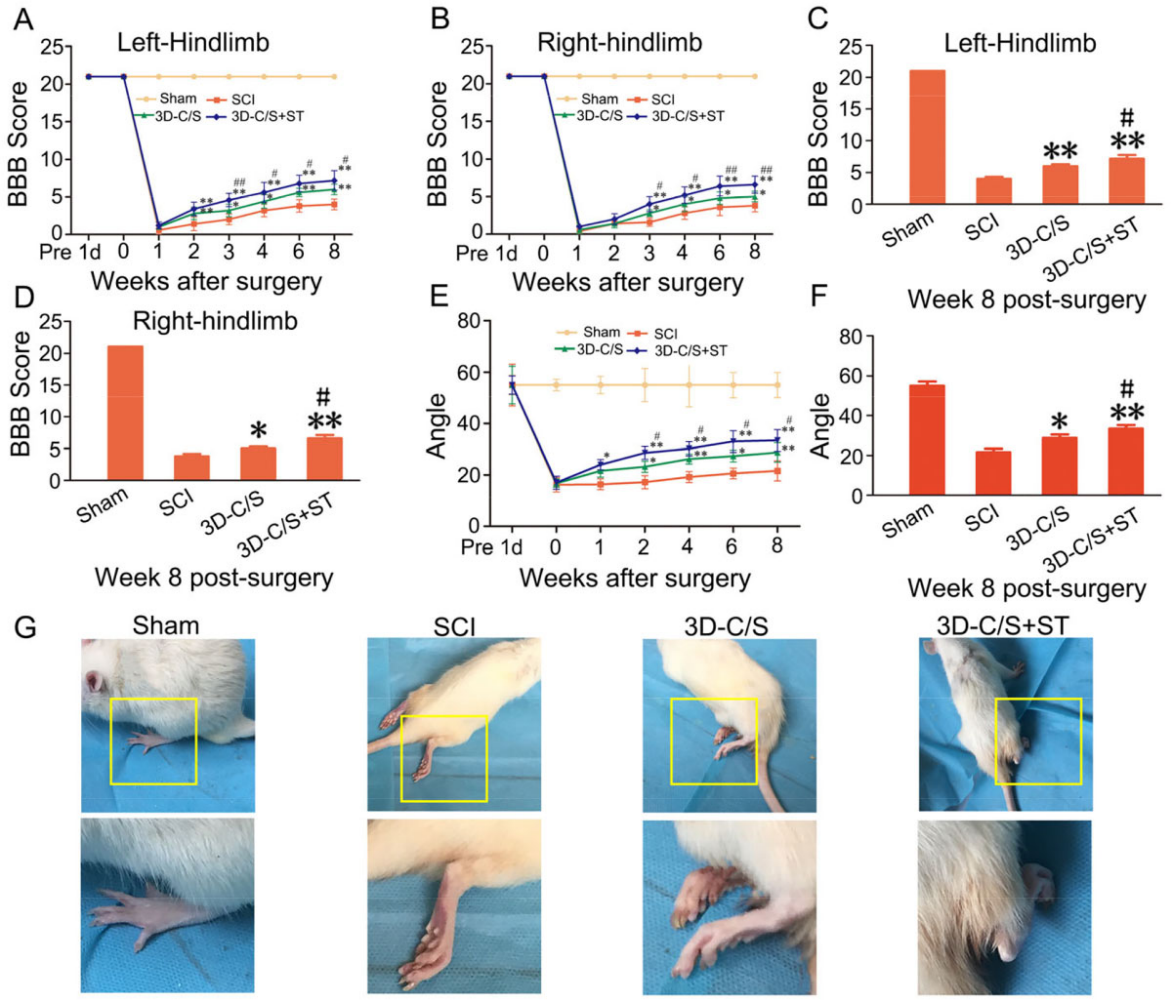
Locomotor function analysis in four groups after surgery. (**A** and **B**) BBB scores of bilateral hindlimbs. (**C** and **D**) BBB scores of bilateral hindlimbs at 8 weeks after surgery. (**E**) The inclined-grid climbing experiment in each group. (**F**) The inclined-grid climbing experiment at 8 weeks after surgery. (**G**) Representative gait records of walking gaits of hindlimbs of rats in each group at 8 week after surgery, the yellow box regions in the image above were enlarged in the image below. *P <* *0.05, **P <* *0.01 vs SCI. ^#^*P *<* *0.05, ^##^*P *<* *0.01 vs 3D-C/S.

Three to eight weeks after surgery, BBB scores of the left and right hindlimbs in the 3D-C/S+ST group were significantly higher than those of the SCI group and 3D-C/S group (*P *<* *0.05) (Fig. 4A and B). Eight weeks after surgery, the 3D-C/S+ST group showed a significantly increased BBB scores compared with SCI group (*P *<* *0.01) and the 3D-C/S group (*P *<* *0.05) (Fig. 4C and D). Consistent with the BBB scoring experiment, the results of the inclined-grid climbing experiment indicated that the slope angle was increased in the 3D-C/S+ST group compared to SCI group and the 3D-C/S group (Fig. 4E). Furthermore, implanting 3D-C/S+ST exhibited a significant increase in the slope angle compared to implanting 3D-C/S (*P *<* *0.05) or without implanting scaffolds (*P *<* *0.01) at 8 weeks after surgery (*P *<* *0.05) (Fig. 4F). Compared with SCI group and 3D-C/S group, the hindlimb walking ability of 3D-C/S+ST group showed significantly better recovery (Fig. 4G).

**Figure 5.**
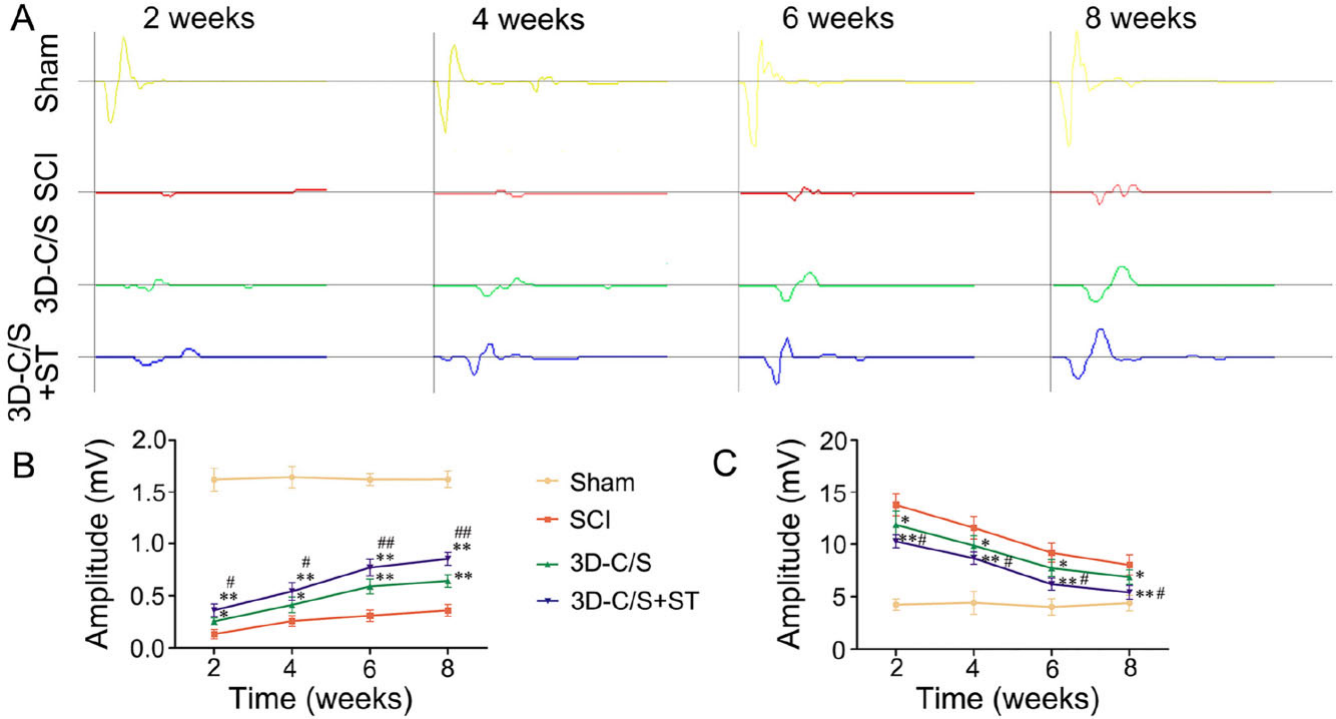
Electrophysiological activity in four groups after surgery. (**A**) Typical recorded MEP traces of hindlimb in each group. (B-C) Quantification of the amplitude (B) and the latency (C) of MEP in each group. *P <* *0.05, **P <* *0.01 vs SCI. ^#^*P *<* *0.05, ^##^*P *<* *0.01 vs 3D-C/S.

Two, 4, 6 and 8 weeks after surgery, the amplitude and the latency of MEP showed improvement in the SCI group, the 3D-C/S group and the 3D-C/S+ST group (Fig. 5A–C). Eight weeks after surgery, 3D-C/S+ST group (amplitude: 0.85 ± 0.06 mV; latency: 5.41 ± 0.65 ms) enhanced the amplitude and reduced the latency compared to the SCI group (amplitude: 0.36 ± 0.06 mV, *P *<* *0.01; latency: 8.03 ± 0.96 ms, *P *<* *0.01) and the 3D-C/S group (amplitude: 0.64 ± 0.06 mV, *P *<* *0.01; latency: 6.87 ± 0.69 ms, *P *<* *0.05) (Fig. 5A–C). The amplitude and latency at 2, 4 and 6 weeks post-surgery showed statistical differences similar to those at 8 weeks post-surgery (Fig. 5A–C).

### MRI and DTI indicated implanting 3D-C/S+ST enhanced nerve fibers tracts regeneration after SCI

**Figure 6.**
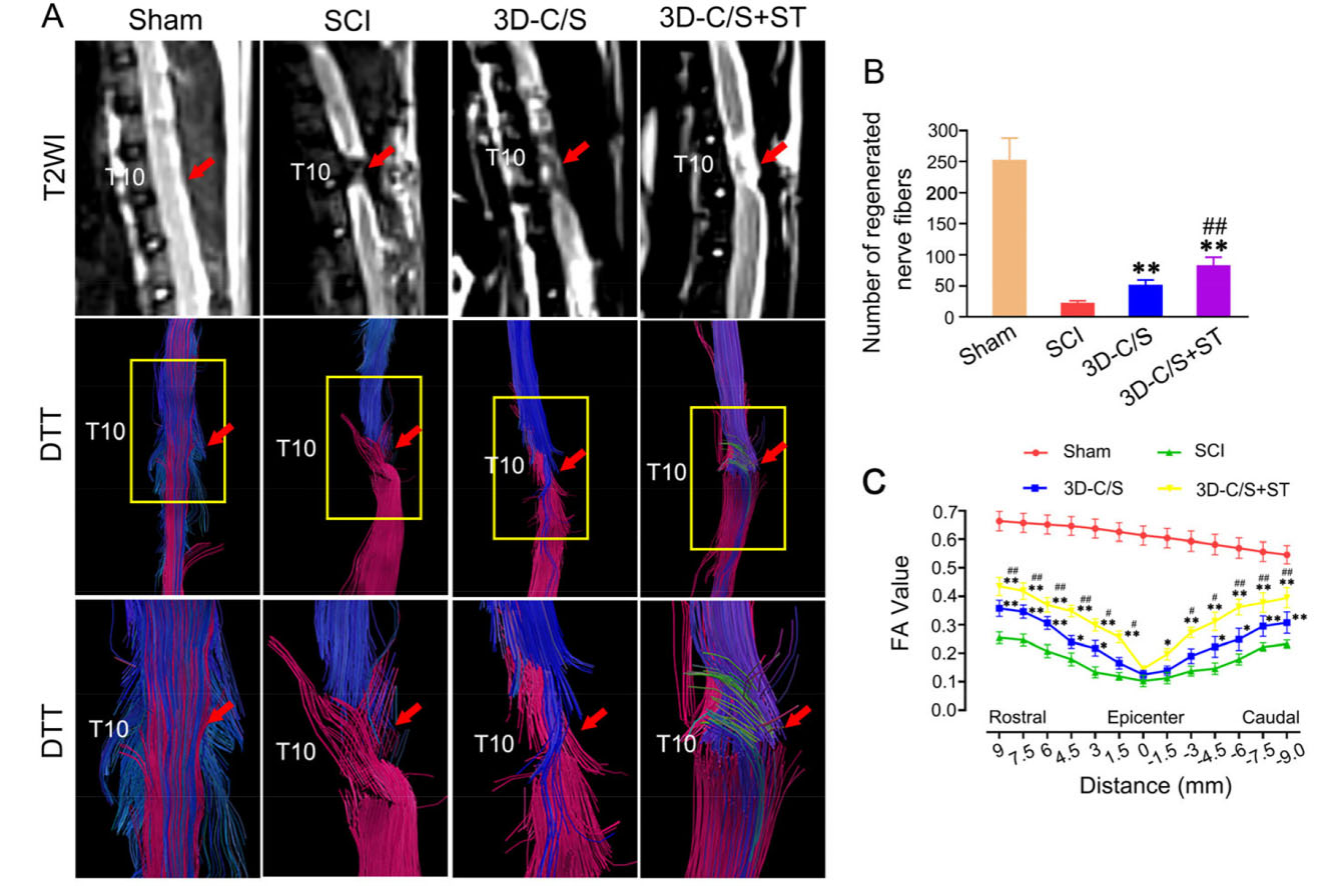
MRI and DTI at 8 weeks after surgery. (**A**) Typical MRI T2 images and DTT images. The yellow box regions in the Middle image were enlarged in the below image. (**B**) Quantification of regenerated nerve fibers at the injury area. (**C**) Distribution map of FA value corresponding to the injury area. *P <* *0.05, **P <* *0.01 vs SCI. ^#^*P *<* *0.05, ^##^*P *<* *0.01 vs 3D-C/S.

Eight weeks after surgery, rats received the implantation of 3D-C/S+ST exhibited more connections at the injury area by T2 weighted imaging (T2WI )and more regenerative nerve fiber tract at the injury area, when compared with those received 3D-C/S and those that did not received any scaffolds (Fig. 6A). After implantation of 3D-C/S+ST for 8 weeks, the number of regenerated nerve fibers (83 ± 13.166) at the injury area was markedly more than that in the SCI group (23.26 ± 3.017, *P *<* *0.01) and the 3D-C/S group (52.37 ± 7.158, *P *<* *0.01) (Fig. 6B). These results indicated that implantation of 3D-C/S+ST might contribute to the regeneration of nerve fibers tracts in the host spinal cord.

There were prominently larger FA values at these locations (9 mm, 7.5 mm, 6 mm, 4.5 mm, 3 mm, 1.5 mm, –3 mm, –4.5 mm, –6 mm, –7.5 mm and –9 mm from the epicenter of the injury site) in the in the 3D-C/S group than in the SCI group and the 3D-C/S group (*P *<* *0.05) (Fig. 6C). Consistent with the results of the MRI and DTT images, the 3D-C/S+ST group exhibits better repair than the SCI group and the 3D-C/S group at these locations (9 mm, 7.5 mm, 6 mm, 4.5 mm, 3 mm, 1.5 mm, –3 mm, –4.5 mm, –6 mm, –7.5 mm and –9 mm from the epicenter of the injury site) based on the FA value.

### The implantation of 3D-C/S+ST markedly reduced cavity formation, facilitate nerve fiber regeneration and remyelination at the injury site

**Figure 7.**
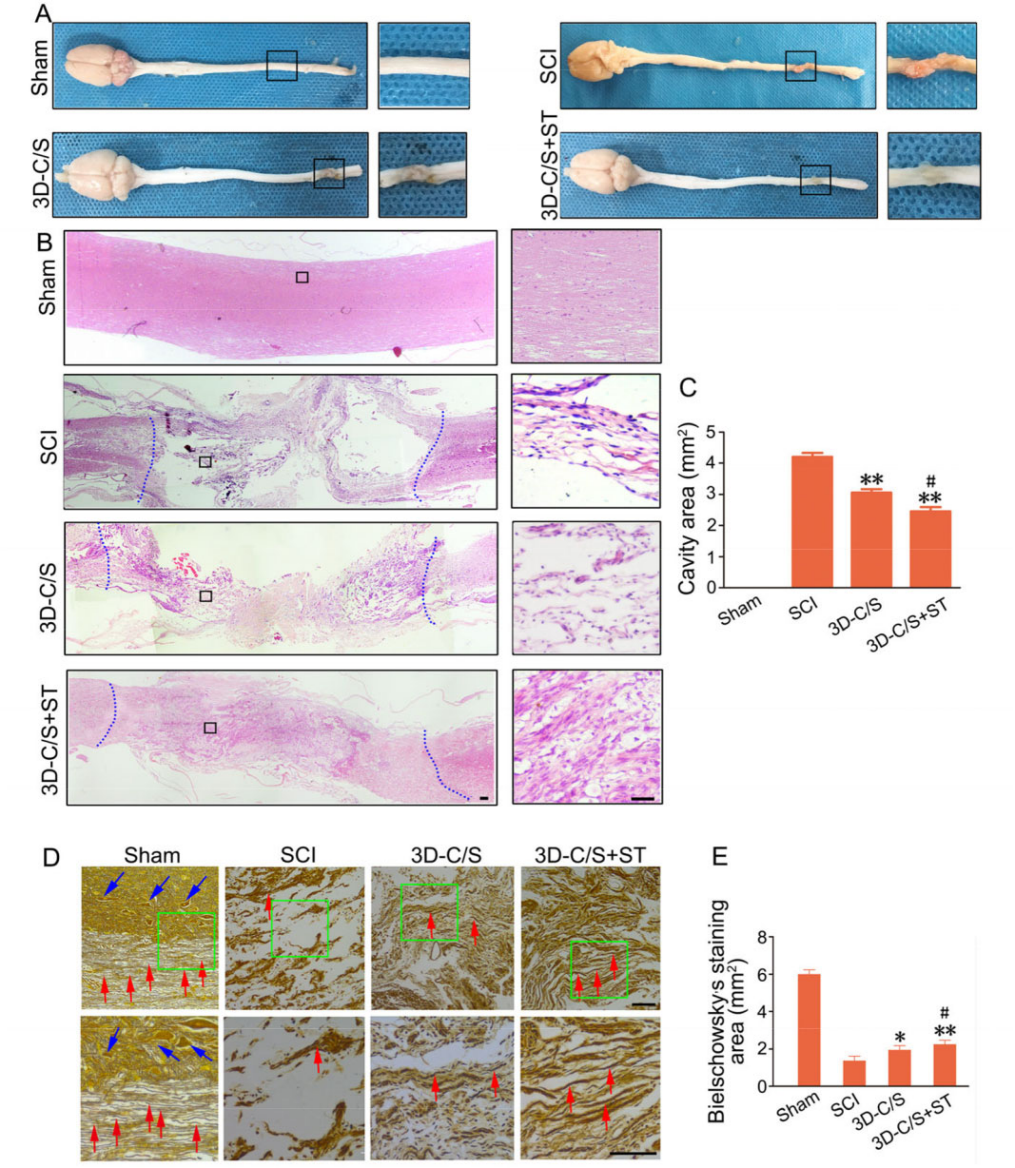
Histology and quantification at 8 weeks after surgery. (**A**) Representative macrograph of spinal cord. The black box regions in the left image were enlarged in the right image. (**B**) Representative HE staining images. The black box regions in the left image were enlarged in the right image. (**C**) Quantification graphs of the average cavity area for HE staining image in rats with different treatments. (**D**) Bielschowsky’s silver staining of spinal cords horizontal sections. The green box regions in the image above were enlarged in the image below. (**E**) Quantification graphs of average Bielschowsky’s silver staining area. *P <* *0.05, **P <* *0.01 vs SCI. ^#^*P *<* *0.05 vs 3D-C/S+ST. Scale bars = 50 µm in panels (B and D).

**Figure 8.**
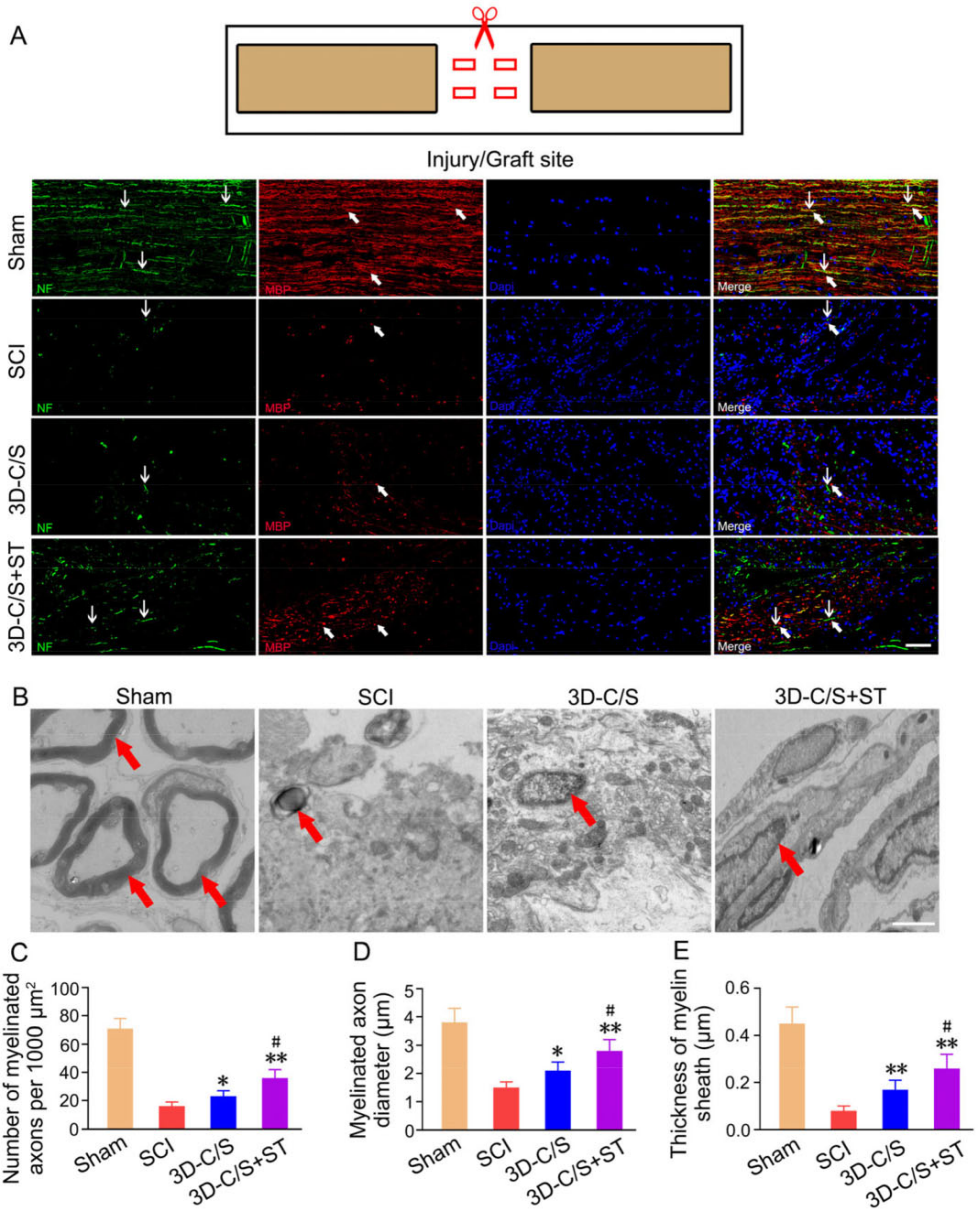
Myelination and the TEM images of the injury site of spinal cord tissue in the Sham, SCI, 3D-C/S and 3D-C/S+ST groups. (**A**) Typical micrographs of NF (green, white arrow) and MBP (red, white arrow) expression. (**B**) Typical TEM images. Myelinated nerve fibers were indicated by red arrows. (**C**–**E**) Quantification of the number of myelinated axons (C), myelinated axons diameter (D) and thickness of myelin sheath (E). *P <* *0.05, **P <* *0.01 vs SCI. ^#^*P *<* *0.05 vs 3D-C/S. Scale bars = 50 µm in panel (A), 1 µm in panel (B).

The improvement of locomotor function may be related to the histology of the SCI site. Histological analysis of the injured spinal cord was performed at 8 weeks postoperatively. Macroscopic observation indicated that the damaged area of 3D-C/S+ST group exhibited better connection and integration with host tissue than the SCI group and the 3D-C/S group (Fig. 7A). The average cavity area were markedly reduced in the 3D-C/S+ST group (2.458 ± 0.429 mm^2^) compared with the SCI group (4.202 ± 0.406 mm^2^) (*P *<* *0.01) and the 3D-C/S group (3.059 ± 0.356 mm^2^) (*P *<* *0.05) (Fig. 7B and C), which suggested implanting 3D-C/S+ST provided tissue support that integrated well with both ends of the SCI area. Implanting 3D-C/S+ST (2.26 ± 0.22 mm^2^) exhibited a significant increase in the Bielschowsky’s silver staining area at the injury site compared to implanting 3D-C/S (1.96 ± 0.23 mm^2^) (*P *<* *0.05) or without implanting scaffolds (1.38 ± 0.24 mm^2^) (*P *<* *0.01) at 8 weeks after surgery (Fig. 7E), suggesting that implanting 3D-C/S+ST could facilitate nerve fiber regeneration at the injury site. The implantation of 3D-C/S+ST markedly increased the number of NF-positive nerve fibers wrapped by MBP-positive myelin sheath structures at the injury site compared to the implantation of 3D-C/S or without any implantation (Fig. 8A), which indicated that implanting 3D-C/S+ST could facilitate nerve fiber regeneration and remyelination at the injury site. At 8 weeks after surgery, rats that received the implantation of 3D-C/S+ST significantly exhibited more myelinated axons, bigger diameter of myelinated axons and thicker myelin sheath at the injury site by transmission electron microscopy (TEM), when compared with those received 3D-C/S and those that did not received any scaffolds (Fig. 8B–E), which suggesting that implanting 3D-C/S+ST could facilitate remyelination at the injury site.

### The implantation of 3D-C/S+ST could increase the formation of synaptic connections at the injury site

**Figure 9.**
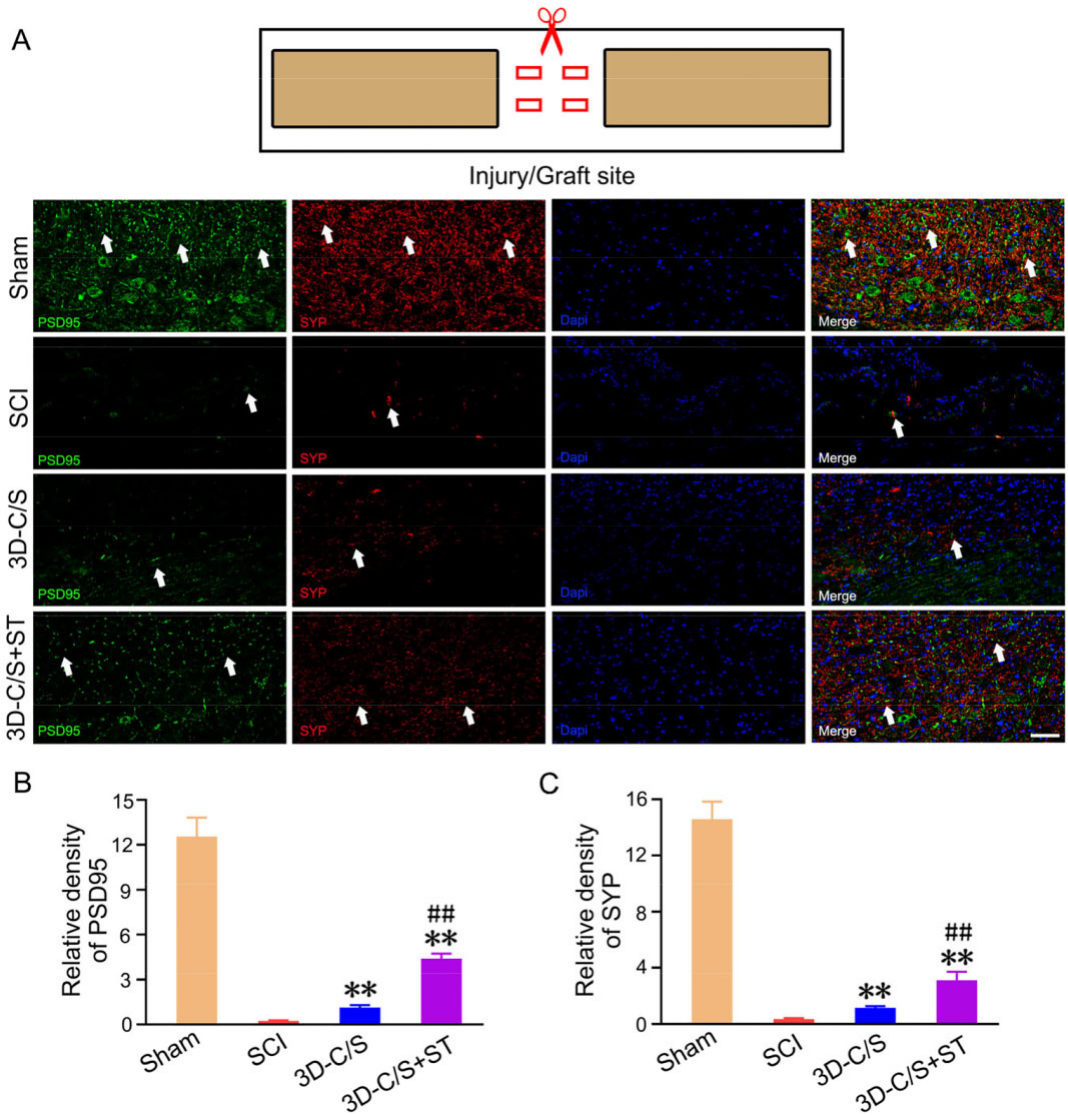
Synaptic connections formation of the injury site of spinal cord tissue on Week 8 post-surgery. (**A**) Representative micrographs of PSD95 (green, white arrow) and SYP (red, white arrow) expression. (**B** and **C**) Quantification graphs of PSD95 (B) and SYP (C) of the injury site of spinal cord tissue. **P <* *0.01 vs SCI. ^##^*P *<* *0.01 vs 3D-C/S. Scale bars = 50 µm in panel (A).

Double-labeled immunofluorescence staining of PSD95 and SYP at the injury/graft site were performed to assess whether implanting 3D-C/S+ST increase the formation of synaptic connections at the injury site at 8 weeks after SCI. The expression of PSD95 and SYP of the 3D-C/S+ST group was significantly higher compared with SCI group (*P *<* *0.01) and 3D-C/S group (*P *<* *0.01), which suggested that implanting 3D-C/S+ST could facilitate the formation of synaptic connections at the injury site (Fig. 9A–C).

## Discussion

Increasing studies demonstrated that the ST derived from MSCs can be an ideal substitute for MSCs transplantation. ST derived from HUCMSCs contains multiple proteins. Therefore, we hypothesized that the ST could have a good therapeutic potential on the treatment of SCI. In this study, we fabricated 3D printed collagen/silk fibroin scaffolds adsorbed with ST of HUCMSCs and implanted the scaffolds into the lesioned area of the rat spinal cord in the rats with complete spinal cord transection. The results showed that compared with 3D-C/S, 3D-C/S+ST could further promote neural regeneration and correspondently ameliorate locomotor function. The benefits of the ST for SCI repair were related to those 79 proteins.

The poor spontaneous regeneration ability after SCI is attributed to many complex biological processes. Microenvironment formed after SCI inhibits effective axon regeneration [67]. After complete spinal cord transection, the loss of all motor and sensory axonal tracts eliminates the possibility of lesion regeneration and recovery within a week, which is a huge challenge in current treatment [68]. The combination of biomaterials and neurotrophic factors can exert their respective advantages and provide excellent favorable effects.

Tissue engineering scaffolds should exhibit favorable biocompatibility, proper mechanical properties and good neuroinduction [69]. Collagen is widely used in the treatment of SCI thanks to its good cytocompatibility. Dai demonstrated that collagen scaffolds combined with neurotrophic factors could facilitate nerve regeneration after SCI in rats and Beagle dogs [70, 71]. However, the poor biomechanics and rapid degradation *in vivo* of collagen limit its use. Silk fibroin possesses some advantages, such as good biocompatibility, stable structure, high mechanical properties and slow degradation *in vitro*. Therefore, the combination of collagen and silk fibroin can greatly reduce each other’s shortcomings. An increasing number of studies indicated that MSCs and their ST can ameliorate the microenvironment of neurogenesis [72]. The growth factors of the ST have been shown to have a significant effect on neuron survival, differentiation and neurite growth [29, 38, 73]. The results of the cytokine antibody array demonstrated that ST derived from HUCMSCs contained 79 proteins among the 174 proteins analyzed. Treatment of CNS disease in the ST is related to multiple factors rather than a single factor. In our study, we fabricated porous 3D printed collagen/silk fibroin scaffold adsorbed with ST. The infrared spectroscopy data revealed that 3D-C/S+ST had suitable fat-soluble and water-soluble chemical bonds that were suitable for the adhesion and growth of nerve cells, hydrogen bonds between collagen/silk fibroin and ST may be formed. The results of DSC indicated that the interaction between ST and 3D printed collagen/silk fibroin might augmented the stability of ST. For X-ray diffraction analysis, 3D-C/S+ST owned ideal crystallinity, which facilitated the formation of the stability of the scaffold and the control of the degradation rate. After co-culturing with NSCs, the 3D-C/S+ST showed favorable cytocompatibility.

We established a rat T10 complete spinal cord transection model [74] and used BBB scores, inclined-grid climbing test, MEP, MRI and DTT to estimate neuroregeneration and function recovery. The results of behavioral assessment and electrophysiological studies could reveal the recovery of the 3D-C/S+ST group was significantly improved compared to SCI group and 3D-C/S group, suggesting that 3D-C/S+ST was conducive to the reconstruction of locomotor function after SCI. Locomotor function recovery was consistent with changes in imaging. MRI qualitatively describes the morphological changes of the tissue [75, 76], while DTI is a quantitative observation of the microstructure of the tissue. The combination of MRI and DTI in this study provides solid data to estimate repairing after SCI. FA values can reveal white matter integrity, such as damage to axon structures and demyelination. In our study, high FA values reflect favorable repair after SCI. DTT can track local fibers in the area of SCI and visualize the morphological changes of the spinal cord. Compared with traditional MRI, DTI is more sensitive to detect the repair of SCI. The results of MRI and DTI indicated the repair of SCI was markedly enhanced in the 3D-C/S+ST group compared to SCI group and 3D-C/S group. Histological analysis could provide more precise evidence for locomotor function repair. For the macroscopic histology images HE staining, we found implanting 3D-C/S+ST could markedly reduce cavity formation and facilitate spinal tissue regeneration. Bielschowsky’s silver staining revealed that implanting 3D-C/S+ST could further promote the regeneration of spinal nerve fibers compared to implanting 3D-C/S, indicating the ST derived from HUCMSCs created a favorable microenvironment for the repair of SCI. As a critical protein on the myelin sheath, MBP is a necessary structure for nerve impulses to conduct along axons. Immunofluorescence staining showed that the implantation of 3D-C/S+ST significantly increased NF-positive nerve fibers and NF-positive nerve fibers associated with myelin sheath at the injury/graft site, suggesting the implantation of 3D-C/S+ST could provide a suitable microenvironment to facilitate the regeneration of nerve fibers and myelin sheath. TEM analysis indicated transplantation of 3D-C/S+ST enhanced remyelination at the injury site. PSD95 is a post-synaptic marker and SYP is a presynaptic marker. The implantation of 3D-C/S+ST promoted the establishment of synaptic connections at the injury site, which is anatomically crucial for the recovery of neurological function.

However, our study has some limitations. Many functions of 3D-C/S+ST have not been explored in our study. Therefore, we need to investigate whether 3D-C/S+ST can promote cell differentiation, remove reactive oxygen species and reduce inflammation in the injured microenvironment in the future.

## Conclusion

We demonstrated that 3D printed collagen/silk fibroin scaffolds carrying MSCs ST ameliorated neurological dysfunction by bridging spinal cord lesions to partially reconstruct neuronal circuits after SCI in rats, suggesting the implantation of 3D printed collagen/silk fibroin scaffolds carrying MSCs ST have the potential to become a novel and safer treatment for SCI repair.

## Funding

This work was supported by the National Key Research and Development Plan of China (2021YFF1200800), the National Nature Scientific Fund of China (82171861, 81771352, 81971782, 81671222 and 81771350) and the Nature Scientific Fund of Tianjin (18JCJQJC48500 and 19JCYBJC27900).

*Conflict of interest statement*. There are no actual or potential conflicts of interest.
